# Identification of Potential Biomarkers for Group I Pulmonary Hypertension Based on Machine Learning and Bioinformatics Analysis

**DOI:** 10.3390/ijms24098050

**Published:** 2023-04-28

**Authors:** Hui Hu, Jie Cai, Daoxi Qi, Boyu Li, Li Yu, Chen Wang, Akhilesh K. Bajpai, Xiaoqin Huang, Xiaokang Zhang, Lu Lu, Jinping Liu, Fang Zheng

**Affiliations:** 1 Center for Gene Diagnosis, Department of Clinical Laboratory Medicine, Zhongnan Hospital of Wuhan University, Wuhan 430071, China; huhui122109@163.com (H.H.);; 2 Department of Cardial Surgery, Zhongnan Hospital of Wuhan University, Wuhan 430060, China; 3 Department of Genetics, Genomics and Informatics, University of Tennessee Health Sciences Center, Memphis, TN 38163, USA; 4 Department of Ophthalmology, University of Tennessee Health Science Center, Memphis, TN 38163, USA

**Keywords:** Group I pulmonary hypertension, machine learning, pathway enrichment analyses, protein–protein interaction, ferroptosis, immune infiltration, biomarker

## Abstract

A number of processes and pathways have been reported in the development of Group I pulmonary hypertension (Group I PAH); however, novel biomarkers need to be identified for a better diagnosis and management. We employed a robust rank aggregation (RRA) algorithm to shortlist the key differentially expressed genes (DEGs) between Group I PAH patients and controls. An optimal diagnostic model was obtained by comparing seven machine learning algorithms and was verified in an independent dataset. The functional roles of key DEGs and biomarkers were analyzed using various in silico methods. Finally, the biomarkers and a set of key candidates were experimentally validated using patient samples and a cell line model. A total of 48 key DEGs with preferable diagnostic value were identified. A gradient boosting decision tree algorithm was utilized to build a diagnostic model with three biomarkers, PBRM1, CA1, and TXLNG. An immune-cell infiltration analysis revealed significant differences in the relative abundances of seven immune cells between controls and PAH patients and a correlation with the biomarkers. Experimental validation confirmed the upregulation of the three biomarkers in Group I PAH patients. In conclusion, machine learning and a bioinformatics analysis along with experimental techniques identified PBRM1, CA1, and TXLNG as potential biomarkers for Group I PAH.

## 1. Introduction

Pulmonary arterial hypertension (PAH) refers to a progressive pulmonary vascular disease, characterized by pulmonary vascular remodeling and an increased pulmonary vascular resistance, and is associated with a high mortality [1,2]. Group I PAH is the most vital of all pulmonary hypertension subtypes, because of its aggressiveness, restricted therapeutic option, and dismal prognosis. As great efforts have been made in the past 30 years, the survival rate of Group I PAH patients has increased, owing to improved medical services; however, the underlying molecular mechanisms are still unknown [3]. Recently, PAH research has focused on investigating novel diagnostic biomarkers and therapeutic targets. For instance, Nies et al. found that the Insulin-like growth factor (IGF) axis could serve as a diagnostic biomarker for severe pulmonary hypertension [4]. Similarly, Yang et al. uncovered mitogen-activated protein kinase 6 (MAPK6) to be an important gene for discriminating IPAH from healthy controls [5]. A few biological processes have been reported to participate in developing Group I PAH; however, the search of biomarkers associated with this disease needs further exploitation.

The Gene Expression Omnibus (GEO) database-based mining of bioinformatics information is accessible and is useful to identify possible disease pathogenic genes, further paving the way for subsequent studies [6]. In recent years, an increasing amount of research has applied machine learning (ML) techniques that are widely used in disease diagnosis [7] and genomics [8]. ML refers to a computer science branch, which enables computers to “learn” based on training data and make predictions or decisions without being explicitly programmed [9]. ML is broadly classifiable into “supervised” and “unsupervised” learning. The supervised learning (e.g., decision trees, support vector machines) is akin to a type of model fitting with reference to the final outcome, whereas unsupervised learning (e.g., k-means clustering) attempts to identify natural relationships within the data without reference to any outcome [9]. Xiao et al. used different ML models for cancer prediction based on RNA-seq data from diverse cancer datasets and found that the deep-learning-based multimodel ensemble methods had better performance on all the datasets [10]. Liu et al. applied multiple ML models to distinguish between T-cell-mediated rejection (TCMR) and stable function (STA) samples based on RNA-seq data and clinical variables and reported that the random forest (RF) achieved the best performance [11]. Alanni et al. proposed a gene selection method using SVM for classifying cancer types based on microarray datasets [12]. The gene set selected by SVM showed a superior performance in cancer classification compared to that selected by other selection methods. Dai et al. found glaucoma diagnostic markers based on a logistic regression-RF (LR-RF) model coupled with experimental validation [13]. However, no study has been carried out using ML for PAH diagnosis and potential biomarker discovery and further validated the results using a pathway analysis and experiments.

In the current study, Group I PAH-associated molecular biomarkers were identified using seven machine learning methods. The underlying biological significance as well as the immune cell infiltration, potential transcription factor (TF) binding sites, and therapeutic targets relevant to the identified biomarkers were explored using various bioinformatics approaches. Moreover, based on the results from the functional analysis, the key ferroptosis-related genes (FRGs), which were closely associated with these key biomarkers were determined. Finally, the differential expressions of the potential biomarkers were confirmed in clinical tissue samples at the mRNA and protein levels.

## 2. Results

### 2.1. Differentially Expressed Genes between PAH Patient and Control Samples

**Figure 1:**
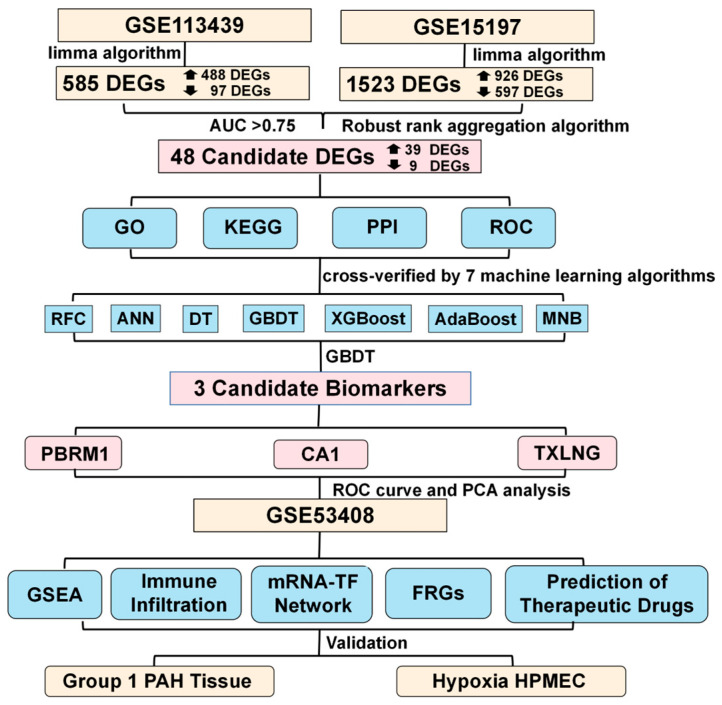
Workflow of the study design. DEGs: differentially expressed genes; AUC: area under the curve; GO: Gene Ontology; KEGG: Kyoto Encyclopedia of Genes and Genomes; PPI: protein–protein interaction; ROC: receiver operator characteristic; RFC: random forest classifier; ANN: artificial neural network; DT: decision tree; GBDT: gradient boosting decision tree; XGBoost: extreme gradient boosting; AdaBoost: adaptive boosting; MNB: multinomial naïve Bayes; GSEA: gene set enrichment analysis; TF: transcription factor; FRGs: ferroptosis-related genes; PCA: principal component analysis; PAH: pulmonary arterial hypertension; HPMEC: human pulmonary microvascular endothelial cells.

**Figure 2:**
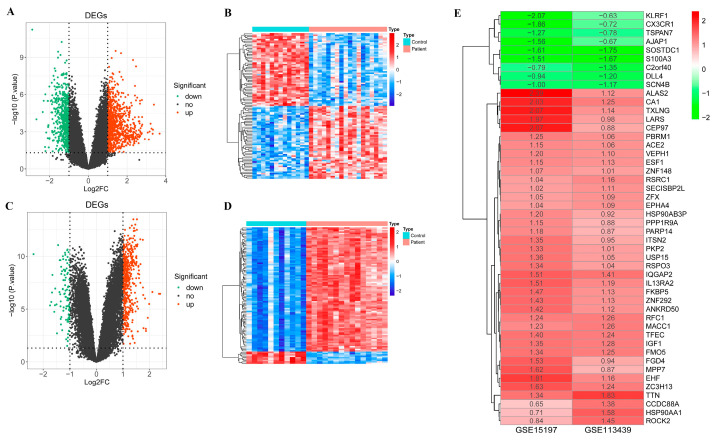
Screening of key differentially expressed genes (DEGs) between PAH patient and control groups. (**A**) Volcano plot showing DEGs obtained in the GSE15197 dataset, with red and green colors indicating up- and downregulated genes, respectively. (**B**) Heatmap showing the top 100 DEGs with red and blue indicating high and low expression patterns across PAH and control samples in GSE15197. (**C**) Volcano plot showing DEGs with up- (red) and downregulated (green) genes obtained in the GSE113439 dataset. (**D**) Heatmap showing the top 100 DEGs with red and blue colors indicating a high and low expression of the genes, respectively, across PAH patient and control samples in the GSE113439 dataset. (**E**) RRA integration analysis of DEGs with up- (red) and downregulated (green) genes obtained from both GSE15197 and GSE113439 datasets.

The study design followed in the current study is shown in Figure 1. For identifying DEGs between PAH patient and control samples, a differential analysis was performed using the GSE15197 and GSE113439 datasets independently. In the GSE15197 dataset, there were 1523 DEGs between patients and healthy subjects, including 926 upregulated and 597 downregulated genes (Figure 2A). A heatmap of the 100 most significant DEGs showed that almost half of these genes were upregulated in PAH patients (Figure 2B). A total of 585 DEGs (488 upregulated and 97 downregulated) were found between PAH patients and healthy subjects in the GSE113439 dataset (Figure 2C). As shown in the heatmap, most of the top 100 DEGs were highly expressed in the patient group (Figure 2D). Using an RRA integration analysis, 48 DEGs (39 up- and 9 downregulated in patient versus control group) were found to be common in both datasets (Figure 2E) and were considered further.

### 2.2. Candidate DEGs

**Figure 3:**
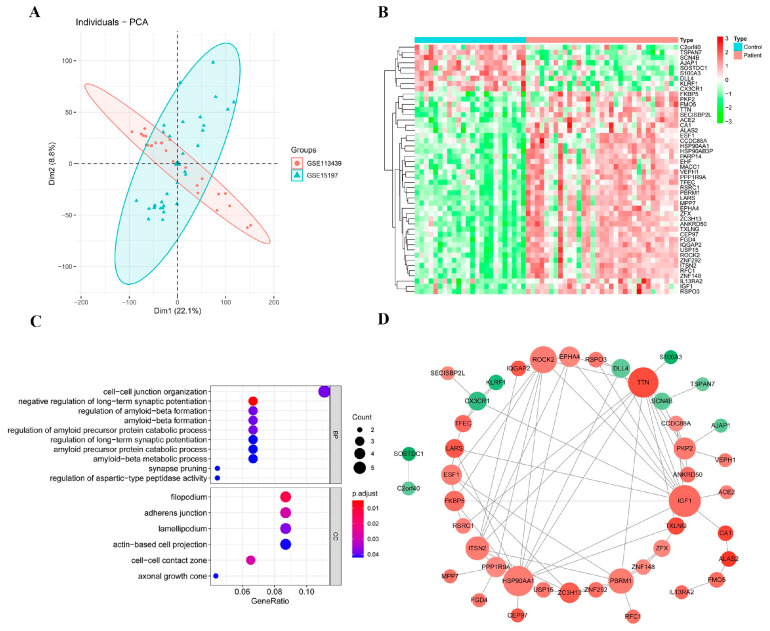
Functional enrichment and PPI network analyses. (**A**) PCA results. The red color circles denote GSE113439 samples and the blue color triangles represent GSE15197 samples. (**B**) The expression pattern of the 48 candidate DEGs. Red color indicates a high expression, whereas green color represents a low expression of the genes across PHA and control samples. (**C**) Bubble plot showing GO enrichment results for the 48 candidate DEGs with x- and y-axes indicating annotation terms and richness factor (DEG number-to-total gene number ratio within one specific term), respectively. Dot color and size indicate adjusted *p*-value range and gene count, respectively. (**D**) PPI network analysis results. The node size indicates the number of interacting partners (larger nodes have more interacting partners). Red color nodes are upregulated, and green color nodes are downregulated.

Our principal component analysis (PCA) results showed insignificant batch effects between both datasets (Figure 3A). In addition, the expression pattern of the resulting candidate DEGs based on both datasets also showed a significant difference between the patient and control groups (Figure 3B). Furthermore, a favorable diagnostic value was obtained for the 48 candidate DEGs by the receiver operator characteristic (ROC) curve analysis (AUC > 0.75) (Table 1).

### 2.3. Functional Enrichment Analysis of Candidate DEGs

The results from the Gene Ontology (GO) and Kyoto Encyclopedia of Genes and Genomes (KEGG) pathway analyses revealed that the DEGs were associated with 19 GO terms, but no KEGG pathways. This could be due to the low number of genes used for the enrichment analysis. The key DEGs were annotated in 10 biological process (BP) terms, including the cell–cell junction organization, the regulation of amyloid-beta formation, and the negative regulation of long-term synaptic potentiation, and six cellular components (CC), such as filopodium, adherens junction, lamellipodium, actin-based cell projection, cell–cell contact zone, and axonal growth cone (Figure 3C).

### 2.4. Protein–Protein Interaction (PPI) Network of Candidate DEGs

Using the STRING database, a PPI network was constructed among the 48 candidate DEGs to identify the most significant clusters and interactions among DEGs (Figure 3D). Most of the candidate genes were found to be interacting with other genes in the network. The average node degree of the PPI network was found to be ~3, while there were a total of 75 connections among the candidate genes. Interestingly, a few genes, such as HSP90AA1, TTN, IGF1, PBRM1, and ROCK2 had a high node degree and interacted with multiple partners in the PPI network. The average log2-fold change (based on both datasets) for these genes was >1.0 in PHA patient samples compared to normal controls. While TTN was upregulated by approximately 1.6-fold, IGF1 and PBRM1 were each upregulated by at least 1.2-fold, and HSP90AA1 and ROCK2 were each upregulated by 1.1-fold in the PHA patient samples. At the same time, many highly differentially regulated genes had a smaller number of connections with other genes, suggesting that these have not been well-studied and demand further investigation.

### 2.5. Diagnostic Models and Potential Biomarkers

In order to obtain the potential biomarkers with diagnostic value, seven machine learning algorithms were implemented using 48 candidate DEGs to determine the accuracy and positive predictive rates.

**Figure 4:**
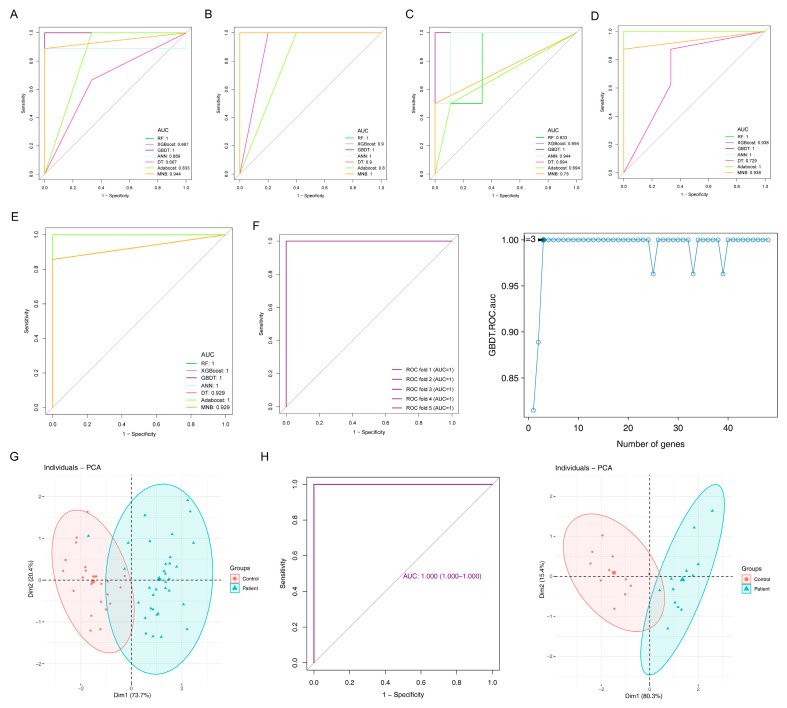
Construction and validation of diagnostic models. (**A**–**E**) The ROC curves for five repeated fivefold cross-validations are shown for individual machine learning algorithms as well as the multivariable model. The area under the ROC curve (AUC) values are shown for each model in the same color as the ROC curve. (**F**) The AUC of the GBDT algorithm was found to be significantly higher than that of the others in fivefold cross-validations (**left**), and the AUC of the GBDT model under different gene numbers is shown (**right**). Gene sets were incorporated in a Bayesian multiple kernel model in line with time order in the case of change point occurrence. The AUC showed an increasing trend first, which peaked at the inclusion of a 3rd gene set, later showing a decreasing trend when up to 25 gene sets were added, followed by another increase. (**G**) PCA analysis of the biomarkers could effectively distinguish normal and disease samples in datasets GSE15197 and GSE113439 after batch correction. (**H**) The ROC curves in the GSE53408 dataset (**left**) and the PCA analysis of the biomarkers could effectively distinguish the normal samples from the case cohorts (**right**).

Following the performance comparison of seven models using five repeated fivefold cross-validation methods, multiple techniques for feature selection were tested and it was found that gradient boosting decision tree (GBDT)-based models gave the best performance in terms of a mean AUC value of one and an accuracy rate of 0.93 compared with the others (Table 2). Hence, GBDT was used to construct the final diagnostic classification model. Extreme gradient boosting (XGBoost) has been used in many high-dimensional datasets and outperformed other models. However, we did not observe this in our study; it might be due to the small dataset that XGBoost did not perform well in the testing set. Figure 4A–E shows the ROC curve of the models in each fold of the testing set. We found the shape of the curve to look rigid, and we double-checked the probability output of the prediction and found that the output of the decision tree model was [0, 1] or [1, 0]. It could be because for a tree-based model, the probabilities are proportional to the class distribution of the samples contained in the leaf; for a single fully grown tree, all terminal nodes are pure, so a leaf contains only one single sample, hence the class “distribution” is [0, 1] or [1, 0]. For the other models, we think it might be due to the small sample size, so the model had a very high/low probability of one class in the testing data, leading to limited thresholds on the curve. However, this did not limit the selection of the optimal model as we considered both AUC and accuracy.

Based on the optimal algorithm, the AUC reached the maximum value when the model included the top three genes, PBRM1, CA1 and TXLNG, which were defined as potential biomarkers of PAH (Figure 4F). The PCA analysis result showed that the biomarkers could effectively distinguish normal and disease samples in the datasets GSE15197 and GSE113439 after batch calibration (Figure 4G). The optimal AUC was one, indicating the excellent performance of the GBDT model with the three genes. On the other hand, this might be because the sample size was small, causing the model to easily distinguish disease and normal samples. To justify the credibility of the marker genes identified by machine learning, we used GSE53408 as an external dataset to validate the model, and the AUC was found to be one when testing on the GSE53408 dataset. The biomarkers could effectively distinguish the control samples from the disease samples (Figure 4H). We further employed a functional enrichment analysis and experimental validations to support the prediction results.

### 2.6. Functional Enrichment Analysis of the Three Biomarkers

**Figure 5:**
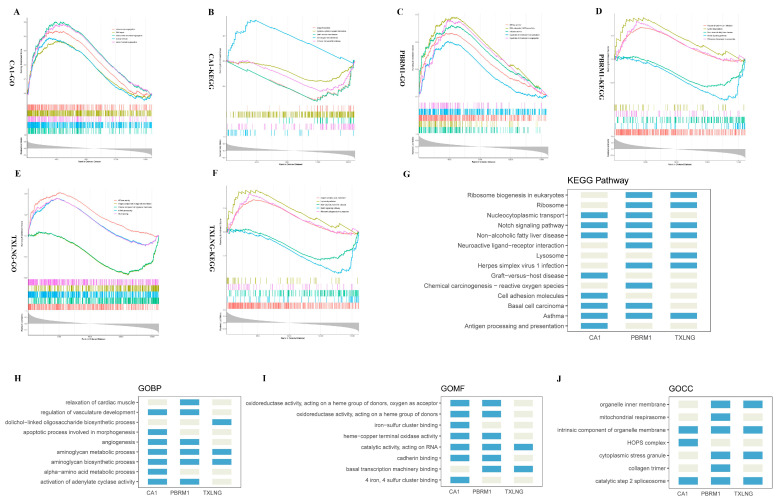
Functional enrichment analysis of the three biomarkers. Top 5 GO annotations and KEGG pathways for (**A**,**B**) CA1, (**C**,**D**) PBRM1, and (**E**,**F**) TXLNG, respectively. (**G**) Summary of CA1, PBRM1, and TXLNG KEGG pathways. (**H**–**J**) Summary of GO annotations for CA1, PBRM1, and TXLNG, respectively. GOBP: GO biological processes, GOMF: GO molecular functions, and GOCC: GO cellular components.

Following the identification of the three biomarkers, a single-gene GSEA was first used for analyzing gene sets with a statistically significant difference between the high-expression and low-expression groups of PBRM1, CA1, and TXLNG. Coexpressed genes associated with these biomarkers were obtained through a Pearson correlation analysis using the GSE113439 and GSE15197 datasets. The top five GO annotations and KEGG signaling pathways are displayed in Figure 5A–F (Supplementary Table S1). The result showed that the three biomarkers were commonly enriched in the Notch signaling pathway, nonalcoholic fatty liver disease, and asthma pathways (Figure 5G). In addition, these biomarkers were commonly annotated to the GO biological processes (GOBP) terms aminoglycan biosynthesis process and aminoglycan metabolic process, the GO molecular functions (GOMF) term catalytic activity acting on RNA, and the GO cellular components (GOCC) terms intrinsic component of organelle membrane and catalytic step 2 spliceosome (Figure 5H–J). Meanwhile, we noticed that the GOMF terms corresponding to the CA1 and PBRM1 genes included ferroptosis-related annotations, such as iron–sulfur cluster binding.

### 2.7. Immune Infiltration and Its Relation to the Biomarkers

**Figure 6:**
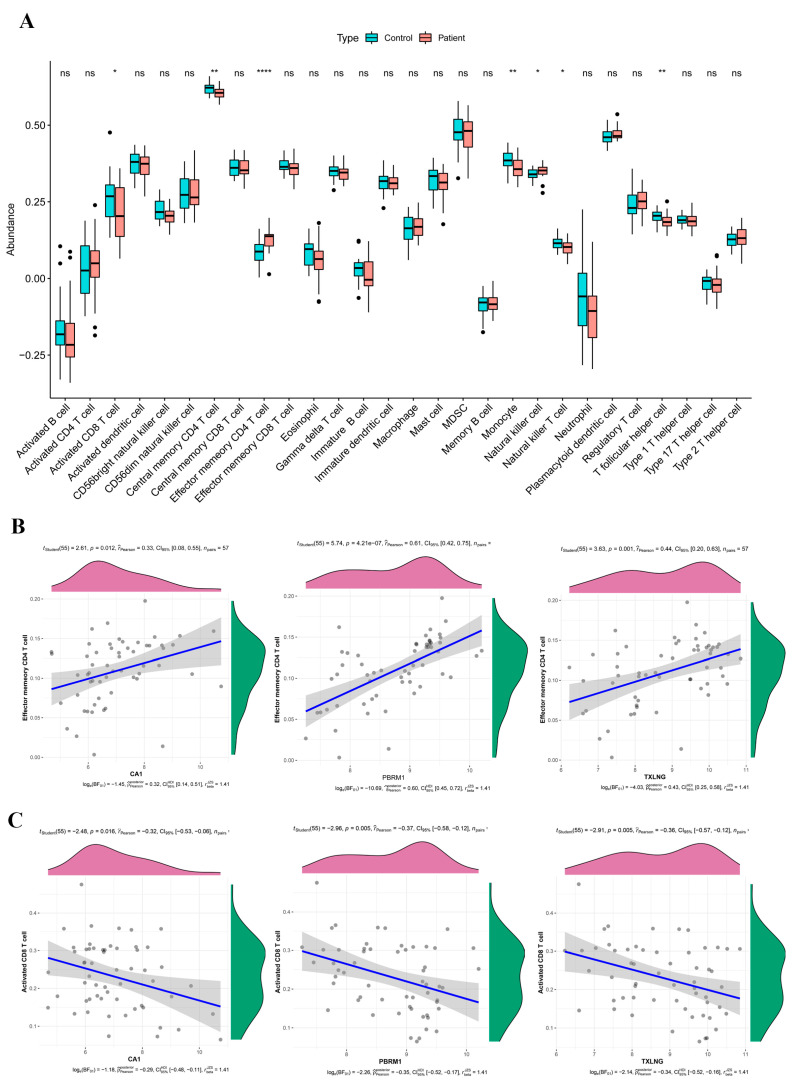
Analysis of immune infiltration. (**A**) Infiltration of different immune cells between Group I PAH patients and normal controls. The red boxplot corresponds to Group I PAH patients, while the blue boxplot corresponds to normal controls. * *p* < 0.05, ** *p* < 0.01, **** *p* < 0.001, ns: not significant. (**B**) Effector memory CD4 T cells showed a positive association with the three biomarkers, whereas (**C**) activated CD8 T cells showed a negative association.

To investigate the relationships between immune cells and the three biomarkers in PAH, an immune infiltration analysis in Group I PAH was conducted As a result, the expression of seven immune cell gene sets (natural killer T cell, activated CD8 T cell, effector memory CD4 T cell, central memory CD4 T cell, T follicular helper cell, natural killer cell, and monocyte) were significantly different between controls and PAH patients (Figure 6A). In addition, a correlation analysis indicated that effector memory CD4 T cells were positively associated with the three biomarkers (Figure 6B), and the correlation between memory CD4 T cell and CA1, PBRM1, TXLNG was 0.33155 (*p* = 0.01170), 0.6120 (*p* = 4.21 × 10^−7^), and 0.43983 (*p* = 0.00061), respectively. Most immune cells showed a negative correlation with the biomarkers (Table 3, Figure 6C).

### 2.8. Regulatory Network of Biomarker-TFs and Predicted Therapeutic Drugs

**Figure 7:**
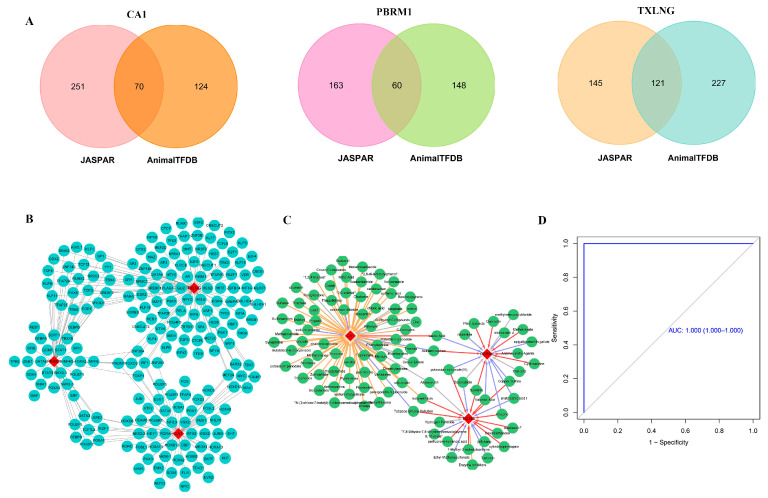
Biomarker-TF regulatory network and prediction of therapeutic drugs. (**A**) TFs targeting the three biomarkers were predicted by JASPAR and AnimalTFDB3.0 databases and were used to construct a regulatory network. A total of 70, 60, and 121 TFs were predicted for CA1, PBRM1, and TXLNG, respectively. (**B**) Network of predicted TFs and candidate genes; blue circles are TFs and red diamonds are candidate genes. (**C**) Prediction of therapeutic drugs targeting the three candidate genes; red diamonds represent the candidate genes, and green circles represent the drugs. Yellow arrows represent drugs that reduce the activity of the gene, purple arrows represent the drugs that inhibit gene expression, red arrows indicate drugs that elevate the expression of the gene, and the gray arrows indicate drugs that have other effects on the genes. (**D**) ROC curve analysis of the ferroptosis-related genes associated with potential biomarkers.

TFs targeting the three biomarkers were predicted and regulatory networks were constructed to understand the regulation underlying the three biomarkers. A total of 70, 60, and 121 TFs were predicted to bind CA1, PBRM1, and TXLNG promoters, respectively (Figure 7A). Among these, six TFs, including PRDM1, FOXO3, FOXP1, IRF1, ZNF263, and ZNF384, targeted all three biomarkers (Figure 7B).

Moreover, the comparative toxicogenomics database (CTD) database was used to explore potential therapeutic drugs targeting the identified biomarkers to provide a reference for the treatment decisions (Table 4). A total of 71 potential drug compounds targeted the CA1 gene, most of which were reported to reduce its activity. Around 19 drugs were retrieved for the PBRM1 gene, and many were found to reduce its expression. The TXLNG gene was targeted by 21 potential drug molecules, a considerable number of which were reported to elevate the expression of this biomarker (Figure 7C).

### 2.9. Correlation between Biomarkers and FRGs

Ferroptosis-related annotations in the single gene enrichment results of CA1 and PBRM1 were noticed. These mainly included annotations, such as clustering on oxygen as acceptor, acting on the heme group of donors, oxidoreductase activity, iron–sulfur cluster binding, 4 iron, 4 sulfur cluster binding, and heme-copper terminal oxidase activity, which is strongly associated with the lipid peroxidation of ferroptosis.

For exploring the significance of ferroptosis-related signals in PAH patients, we explored the functional relationship between 259 FRGs and our 3 biomarkers. There were two FRGs associated with CA1 gene, and nine each associated with PBRM1 and TXLNG genes (correlation coefficient > 0.6, *p* < 0.05, Table 5). The ROC curve analysis indicated that FRGs related to the potential biomarkers could perfectly distinguish PAH samples from the control samples, and these FRGs may have a high diagnostic value for PAH patients (Figure 7D).

### 2.10. Differential Expressions of Potential Biomarkers and FRGs in PAH Were Experimentally Confirmed

**Figure 8:**
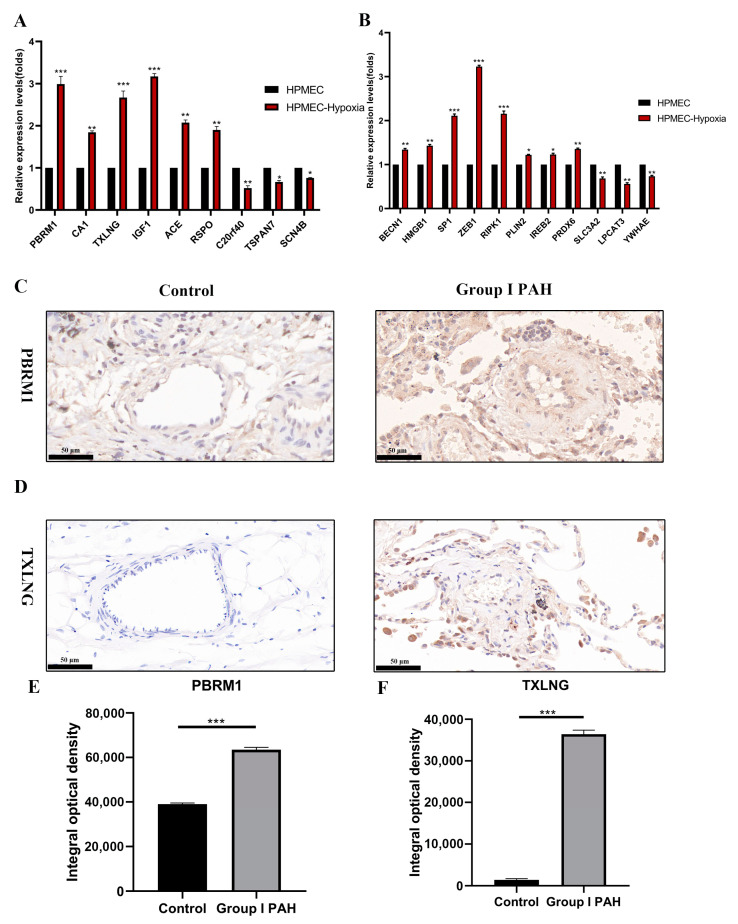
qRT-PCR validation using lung samples and IHC staining of pulmonary microarteries. (**A**) The mRNA expression of seven upregulated (PBRM1, CA1, TXLNG, IGF1, ACE, RSPO) and three downregulated (C2of40, TSPAN7, SCN4B) candidate genes were confirmed by qRT-PCR. All 10 genes were notably differentially expressed in HPMEC under hypoxia for 24 h compared with normoxic control. (**B**) The mRNA expression levels of ferroptosis-related genes (BECN1, HMGB1, SP1, ZEB1, SLC3A2, LPCAT3, RIPK1, PLIN2, IREB2, PRDX6, YWHAE) were confirmed by qRT-PCR. The 11 genes showed remarkable differential expression after a 24 h hypoxia treatment of HPMECs relative to normoxic controls (*n* = 3; * *p* < 0.05, ** *p* < 0.01). Immunohistochemistry (IHC) staining with pulmonary microarteries for (**C**) PBRM1 and (**D**) TXLNG of normal and Group I PAH patients. (**E**,**F**) Typical IHC images obtained at a 400× magnification together with average integral optical density (IOD) were used for analyzing the IHC results of PBRM1 and TXLNG, respectively, in pulmonary control (*n* = 3) and Group I PAH (*n* = 3) samples. Results are the IOD of three visual fields per sample per group (mean ± SD, * *p* < 0.05, ** *p* < 0.01, *** *p* < 0.001).

Finally, the expression of three biomarkers was determined through the qRT-PCR and IHC approaches to validate the accuracy of the results obtained through machine learning and the bioinformatics analysis. A pulmonary hypertension cell model was used to detect the relative mRNA expression of key DEGs including the 3 biomarkers and 11 FRGs. As shown in Figure 8A,B, the key DEGs PBRM1, CA1, TXLNG, IGF1, ACE, and RSPO and FRGs BECN1, HMGB1, SP1, ZEB1, RIPK1, and PRDX6 showed a remarkable upregulation in the 24 h hypoxia-treated HPMEC group relative to the normoxic group. BECN1, HMGB1, SP1, ZEB1, RIPK1, and LPCAT3 had a positive correlation with both PBRM1 and TXLNG, whereas PRDX6 had a positive correlation with TXLNG, and SLC3A2 had a positive correlation with PBRM1. Furthermore, the expression of PBRM1 and TXLNG biomarkers increased during pulmonary vascular remodeling relative to controls at the protein level (Figure 8C–F).

## 3. Discussion

Pulmonary artery hypertension is a highly fatal pathophysiological syndrome featured by pulmonary vascular remodeling and progressively increasing pulmonary vascular resistance, finally causing right heart failure (HF) or even death. As high-throughput technologies have been increasingly applied in PAH, largescale data can be obtained in publicly available databases. Moreover, many studies have been performed to identify molecular biomarkers and explore the underlying mechanisms of PAH based on public data [5,14,15,16]. Despite the recently made great efforts, Group I PAH remains largely unexplored concerning its molecular mechanism and pathogenesis. This is possibly due to the intricate gene mutations and the incapability of conventional PAH cell and animal models to solve such problems.

We started with seven ML methods (RFC, ANN, DT, GBDT, XGBoost, AdaBoost, and MNB) to build an initial diagnostic model. GBDT was chosen to build the final diagnostic model based on the AUC value. Its major idea is that the gradient descent direction of a previous model loss function is built whenever the model is constructed. Model performance can be evaluated by a loss function (in general, it represents the fitting degree and a regular term), with a decreased loss function indicating superior performance. The continuous decline of the loss function can improve the performance of the model, and it is advisable to decrease the loss function along the gradient direction. Gradient boosting is a framework that can fit a number of different algorithms into it and the GBDT algorithm has the following advantages [17]: a high prediction accuracy; suitable for low dimensional data; able to deal with nonlinear data; a flexible processing of diverse data types such as discrete and continuous data; a great accuracy for a low decision time; the use of certain robust loss functions; and robustness to outliers [17]. Here, we processed a total of 48 key DEGs with preferable diagnostic value, compared seven machine learning algorithms, and eventually applied the GBDT machine learning algorithm to build a diagnostic model with three genes, PBRM1, CA1 and TXLNG, that had a significant differential expression between PAH and control samples and were finally regarded as molecular biomarkers of Group I PAH.

PBRM1 encodes BAF180; it is an important subunit of the chromatin remodeling complex SWI/SNF and is related to cell growth, differentiation, as well as DNA repair [18]. PBRM1 has been identified as a recognition factor for the lysine acetylation (K382Ac) of the p53 protein at position 382, specifically through its bromine domain (BD4) and known to be the tumor suppressor of different cancer types. PBRM1 expression or mutation exhibits a diverse significance in predicting the prognosis of different cancers [19]. Huang et al. reported that the ablation of PBRM1 generated an impairment of the epithelial-to-mesenchymal transition (EMT), while arresting epicardium maturation during early development [20]. According to our results, PBRM1 is associated with ferroptosis-related genes. Whether this is the cause of pulmonary hypertension needs to be explored further.

Carbonic anhydrase 1 (CA1) is a zinc enzyme that has an important role in acid–base balance [21]. Fluctuation in carbonic anhydrase expression possibly induces glaucoma, hypertension, neuropathic pain, epilepsy, and cancer [22,23,24,25,26]. Although CA1 is second only to hemoglobin in content in red blood cells, a deficiency of CA1 does not cause blood disease, possibly because other carbonic anhydrases compensate for its deficiency [27]. CA1 is highly expressed and is expected to become a new early diagnostic marker for non-small cell lung cancer (NSCLC) [28,29].

TXLNG is one of the taxilin family members. It has been reported that TXLNG in the cytoplasm may participate in regulating ER stress responses to hypoxia [30,31]. Currently, reports related to TXLNG are limited, and additional studies are needed to explore its detailed functions. In this study, we found that TXLNG was correlated with ferroptosis-related genes. However, further experiments are required to prove its causal role in pulmonary hypertension.

The research related to the identification of new PAH biomarkers generally involves a GSEA analysis of key biomarkers, a TF/miRNA regulatory network and its enrichment analysis, and the expression verification of key genes (PMID: 32886110, PMID: 32849793, PMID: 35710932). In the current study, the GSEA analysis revealed that the three biomarkers were enriched in Notch signaling, iron–sulfur cluster binding, and asthma pathways. To further study the immune cell infiltration in Group I PAH patients, we assessed the levels of 28 immune cells using the ssGSEA algorithm.

The inflammatory basis of PAH (PMID: 29371380; PMID: 31094755; PMID: 34252346) prompted us to mine immune cell targets associated with the identified biomarkers. Our findings revealed that central memory CD4 T cells, activated CD8 T cells, monocyte, effector memory CD4 T cells, T follicular helper cells, natural killer T cells, and nature killer cells were significantly different between controls and Group I PAH patients. While effector memory CD4 T cells were remarkably positively correlated, most of the other immune cells were negatively correlated to the three biomarkers. In recent years, there have been many reports on the association of the immune system with pulmonary hypertension [32]. Nevertheless, the functions of T cell subpopulations have not been determined for Group I PAH. The increased expression and activation of central memory T cells are related to inflammatory and immune responses [33,34]. Type 17 T helper cells can induce a PAH occurrence under chronic hypoxic conditions [35] and decreased levels of NKT cells facilitate the occurrence of systemic sclerosis [36], which is similar to the findings reported in the current work. Therefore, the aberrant T cell subpopulation levels among PAH cases possibly reflected the impaired and exhausted immune system status; however, additional experiments are needed to confirm this hypothesis.

Identifying the underlying molecular mechanisms of TF (dysfunction) is critical for developing tailored regulatory strategies for PAH (PMID: 36684555). Here, we used the AnimalTFDB3.0 and JASPAR databases to predict regulatory transcription factors binding to the promoters of three biomarkers. All three biomarkers were targeted by six common TFs, i.e., PRDM1, FOXO3, FOXP1, IRF1, ZNF263, and ZNF384. PRDM1 encodes a repressor of beta-interferon gene expression and is known to increase during viral infections. This gene has also been studied in the context of pulmonary function. Wang et al. revealed the differential methylation of PRDM1 to be associated with decreased pulmonary function [37]. Both FOXO3 and FOXP1 belong to the forkhead family of transcription factors and are well-known for their regulatory roles in tumorigenesis [38,39]. Nevertheless, the importance of this family has been recently recognized in pulmonary hypertension [40]. In fact, a recent study showed that exposure to trifluoperazine, an antipsychotic drug, was associated with the downregulation of FOXO3 in pulmonary arterial smooth muscle cells (PASMCs) [41], indicating its druggability in PAH. Another study based on genome-wide association data suggested FOXP1 may be a novel therapeutic target for lung disorders [42]. IRF1 encodes a protein that serves as an activator for genes involved in immune responses and it plays a key role in tumor suppression. A study by Bai et al. [43] suggested that IRF1 and IRF8 might be potential regulators of the SPHK1 (the sphingosine pathway promotes vascular remodeling and induces PAH) overexpression gene set signature in human PASMCs. Furthermore, two zinc finger proteins (ZNF263 and ZNF384) were identified to be targeting the PAH biomarkers. While both these proteins have been studied in the context of carcinogenesis, reports related to leukemia are more common for ZNF384 [44,45]. These zinc finger proteins have not been explored in pulmonary hypertension till now and provide an opportunity to be investigated further in PAH.

To explore the potential therapeutic agents targeting the biomarkers, we searched for biomarker-related drugs or molecular compounds. Our results showed that of the three biomarkers, CA1 was the most targeted molecule (by 71 compounds). Acetazolamide was the most potent inhibitor compound for CA1; its inhibitory effect was supported by more than 15 interactions in the CTD database. Acetazolamide has been found to decrease the activity of CA1 by multiple studies [46,47]. The other important compounds against CA1 were indomethacin, propofol, pyrimidines and sulfonamides, and tobacco smoke, the inhibitory activity of each of which was supported by at least two interactions. Bisphenol was the most potent compound targeting TXLNG with two interactions. While bisphenol A has been reported to decrease the expression of TXLNG [48] in mice, and the cotreatment of human primary adipocytes with bisphenol F along with other compounds increases TXLNG expression [49]. Among the compounds targeting PBRM1, valproic acid was the most promising one owing to its interaction with the marker gene, supported by multiple studies. While two studies reported that valproic acid decreased PBRM1 expression [50,51], one study showed a reduced methylation of the biomarker by valproic acid [52].

We observed that CA1 and PBRM1 were associated with pathways such as oxidoreductase, oxygen as acceptor, acting on heme group of donors, 4 iron, 4 sulfur cluster binding, heme-copper terminal oxidase activity, and iron–sulfur cluster binding, showing a strong association of the biomarkers with ferroptosis-related lipid peroxidation. Hence, we analyzed the relationship of our 3 biomarkers with 259 ferroptosis genes. There were nine FRGs related to each of PBRM1 and TXLNG and two FRGs were correlated with CA1. Pulmonary vascular remodeling represents an important pathological characteristic of PAH, and it is characterized by endothelial dysfunction, extracellular matrix accumulation, and the proliferation of medium smooth muscle cells (SMCs), resulting in thickened vascular wall as well as enhanced pulmonary vascular resistance. In pulmonary vascular endothelium, oxidative stress is suggested to suppress the activity of endothelial nitric oxide synthase, reduce nitric oxide content in blood vessels, and induce abnormal EC and SMC proliferation, thus accelerating pulmonary vascular remodeling as well as PAH [53]. Iron in the cytoplasm can be transported to mitochondria and catalyzed by the Fenton reaction and the Harber–Weiss pathway leading to ROS generation. Nikolai et al. found that Fe^2+^ accumulated in in vitro cultured lung vascular endothelial cells changes the cell structure and polarity, resulting in a proinflammatory cell phenotype. In another study, an iron-chelating agent was injected into chronic hypoxic rats, and it was found that the desensitization reduced the right ventricular pressure and pulmonary arteriolar wall thickness [54]. Ferroptosis is a pathological mechanism that can be interfered with and reversed by related drugs; hence, a search for ferroptosis-related genes is of high clinical value in finding better treatment options for Group I PAH patients.

There are several limitations to this study. First, this study did not investigate the functions of diverse T-cell subpopulations during pulmonary vascular remodeling, which should be explored in future studies. Second, the molecular biomarkers of Group I PAH identified in this study are relatively novel, and they have not been studied in the context of pulmonary hypertension, limiting the supporting evidence of these genes in Group I PAH. Therefore, additional in vivo and in vitro experiments are needed for understanding the molecular mechanisms underlying the three biomarkers we identified. CA1 could not be detected by immunohistochemical methods using existing CA1 antibody; hence, alternative methods for detecting CA1 protein need to be established. Third, further studies are warranted to demonstrate the function of the identified hub genes and TFs in Group I PAH vascular remodeling.

## 4. Methods

We used gene expression data from human PAH and normal lung tissues to identify significantly differentially expressed genes between disease and control conditions. Gene enrichment and protein-interaction analyses were performed to explore the functional significance of the differential genes. Further, candidate genes with diagnostic value were identified from a differential list through an area under the receiver operating characteristic (ROC) curve (AUC) analysis. Following this, seven machine learning methods were implemented to screen for potential biomarkers associated with Group I PAH using independent training and validation sets. The identified biomarkers were then validated through a series of bioinformatics-based methods including immune cell infiltration, FRG analysis, TF-regulatory network, and a potential drug candidate analysis. Finally, in vivo and in vitro experiments were conducted to confirm the biomarkers at the mRNA and protein levels in human samples.

### 4.1. Gene Expression Data

The gene expression profiles of *Homo sapiens* lung tissue (including PAH disease and normal control) were selected for this study. The data from nonhuman sources or nonorganization categories were excluded. PAH-related datasets (GSE113439, GSE53408 and GSE15197) were downloaded from the GEO database (https://www.ncbi.nlm.nih.gov/geo, accessed on 8 May 2021). The GSE113439 dataset was available from 11 normal and 15 Group I PAH tissue samples profiled on GPL6244-17930 [55]. The GSE53408 study contained 11 normal and 12 Group I PAH samples based on the GPL6244-17930 platform [56], whereas the GSE15197 dataset was composed of 13 normal (age 60 ± 11, five males and eight females) and 18 Group I PAH samples (age 44 ± 10, seven males and eleven females) that were profiled on GPL6480-9577 [57]. Moreover, we obtained 259 ferroptosis-related genes (FRGs), which included 111 markers, 108 drivers, and 69 suppressors, from the FerrDb database (http://www.zhounan.org/ferrdb/current/, accessed on 8 May 2021) [58].

### 4.2. Screening for Differentially Expressed Genes (DEGs)

Firstly, according to the chip annotation information, the probe names were modified to the corresponding gene names, followed by the removal of the probes with no gene annotation. In addition, the expression values of multiple probes belonging to the same gene were averaged. Differentially expressed genes (DEGs) between PAH and normal control samples in the GSE15197 and GSE113439 datasets were identified by using the R package “Limma” [59] with the significance threshold set as |Log2FC| > 1 and *p*-value < 0.05. Then, the robust rank aggregation (RRA) algorithm was adopted for integrating the ranking list based on the probability model, and the DEGs overlapping in both datasets were selected as candidate DEGs [60].

### 4.3. Functional Enrichment Analysis and Protein–Protein Interaction (PPI) Network Construction

To explore the potential functions and pathways associated with the candidate DEGs, the R package “clusterProlifer” [61] was used to perform Gene Ontology (GO) and Kyoto Encyclopedia of Genes and Genomes (KEGG) pathway analyses. The enrichment analysis was performed using a *p*-value threshold of 0.05 and “Homo Sapiens” as the species, and the results were visualized using bubble diagrams. Additionally, we used the STRING database (https://cn.string-db.org/, accessed on 10 June 2021) to analyze protein interactions among the candidate DEGs with a confidence score of 0.15, and a PPI network was constructed [62]. Cytoscape 3.7.1 software was used for the further analysis and visualization of the PPI network.

### 4.4. Identification of Candidate DEGs with Diagnostic Value

In general, the identification of diagnostic biomarkers requires a considerable number of samples. However, when considered individually, the number of samples in the GSE113439 and GSE15197 datasets was low. To overcome this limitation, we combined both datasets. We utilized the accessory features of the R package “SVA” function for reducing the batch effects between these two datasets [63]. Furthermore, a PCA was used to evaluate the impact of batch effects [64]. Then, the candidate DEGs were analyzed for their diagnostic value by determining the AUC values, and the R package “pROC” was used to draw the ROC curve [65].

### 4.5. Construction and Validation of Diagnostic Model to Screen for Potential Biomarkers

For the derivation of classification models, a total of seven machine learning algorithms (Figure 1), including a random forest classifier (RFC), an artificial neural network (ANN), a decision tree (DT), a gradient boosting decision tree (GBDT), extreme gradient boosting (XGBoost), adaptive boosting (AdaBoost), and multinomial naïve Bayes (MNB) were implemented with 5 repeated fivefold cross-validations for the candidate DEGs with diagnostic value. A RF (randomForest, version 4.6-14) is the typical bagging ensemble algorithm, with a decision tree being the base estimator. An ANN (neuralnet, version 1.44.2) is inspired by the signaling behavior of neurons and is capable of learning valuable patterns from mass data and features [66]. The DT model (party, version 1.3-9) is a predictive tool for categorical and numerical data, which aims to assign samples to specific classes [67]. GBDT (gbm, version 2.1.8) is a tree-based ensemble approach that iteratively builds weak prediction models that seek to minimize the residual, eventually developing a single strong model [68]. XGBoost (xgboost, version 1.4.1.1) has various tunning variables used in cross-validation and regularization and is comparatively fast [69]. As a boosting algorithm, AdaBoost (adabag, version 4.2) is used in conjunction with other learning algorithms to improve performance by combining the output of other “weak learners” into a weighted sum [70], and we used a decision tree as the base model of AdaBoost in this study. MNB (klaR, version 0.6-15) is a simple but very powerful linear classifier and has been very successful in sorting spam and diagnosing diseases [71]. First, the batch-corrected datasets (GSE113439 and GSE15197) were combined and used for model development. The models were trained on the dataset with 57 samples (combined dataset GSE113439 and GSE15197) and 48 features (the candidate DEGs) using a fivefold cross validation. Each model was optimized using grid search by evaluating the average AUC and accuracy. Then, the genes were ranked based on the importance of the candidate DEGs using the optimal algorithm. In order to further select the candidate marker genes, we included the genes based on the rank sequentially and cumulatively into the optimal model and evaluated the AUC of each model with different numbers of genes. Finally, the smallest number of genes with the greatest AUC value was considered as potential biomarkers. Furthermore, for evaluating our model diagnostic performance, the GSE53408 dataset was employed to validate the model. The ability of the identified biomarkers to distinguish disease samples from the normal/control samples was evaluated by a PCA.

### 4.6. Exploration for the Potential Regulatory Mechanisms of Potential Biomarkers through a Series of Bioinformatic Analyses

#### 4.6.1. Single-Gene Gene Set Enrichment Analysis (GSEA)

In order to investigate the significant pathways between the low expression and high expression groups of the potential biomarkers, we conducted a single-gene gene set enrichment analysis (GSEA). The Pearson correlation coefficient between each of three potential biomarkers and the remaining genes was calculated, and significantly correlated genes (*p* < 0.05) were used for the GO and KEGG pathway analysis using the “clusterProfiler” R package with “org.Hs.eg.db” as the database [14].

Furthermore, considering the enrichment of ferroptosis-related pathways in the single-gene analysis results of biomarkers, 259 FRGs, including 111 markers, 108 drivers, and 69 suppressors were obtained from the FerrDb database (http://www.zhounan.org/ferrdb/current/, accessed on 15 June 2021) to further investigate the key FRGs relevant to the potential biomarkers in PAH patients [38]. FerrDb is the first manually curated database to manage and identify markers as well as regulators associated with ferroptosis and related diseases. At the time of the analysis, the database contained 784 ferroptosis-related articles from PubMed, from which 259 regulatory genes had been extracted and collated. A *Pearson correlation* analysis was used for evaluating the relationship between 259 FRGs and 3 biomarkers based on their expression values, and FRGs correlated with *p* < 0.05 and a correlation coefficient >0.6 were selected.

#### 4.6.2. Analysis of Immune Cell Infiltration

The association of the immune system with PAH occurrence has been examined for many years [32]. Furthermore, it has been shown that rats with T-cell deficiency exhibit aggravated PAH following SU5416 injection [72]. To investigate the relationship between new potential biomarkers and immune infiltration, the infiltration states of various immune cells between the case and healthy control samples were explored. On the basis of 28 published immune cell gene sets [73], gene expression profiles after normalization were imported into the ImmuCellAI website (http://bioinfo.life.hust.edu.cn/ImmuCellAI, accessed on 20 July 2021). Then, the abundance of immune cells in the blood of PAH I patients was estimated by a single-sample GSEA (ssGSEA) algorithm, and significant differences were estimated by a Wilcoxon rank test. Further, a *Pearson correlation* analysis was performed between each of the three potential biomarkers and immune cells with significant differences in PAH patients compared to controls to screen the key biomarker-related immune cells.

#### 4.6.3. Construction of Biomarker-Transcription Factor Regulatory Network

The TF binding sites potentially regulating biomarkers were uncovered to construct a transcriptional regulatory network for understanding the regulatory mechanisms underlying the potential biomarkers. The promoter sequences of the biomarkers (2000 bp upstream of the gene) were downloaded from the NCBI database (https://www.ncbi.nlm.nih.gov/, accessed on 22 July 2021), and the JASPAR (https://jaspar.genereg.net/, accessed on 22 July 2021) and AnimalTFDB3.0 (http://bioinfo.life.hust.edu.cn/AnimalTFDB/, accessed on 22 July 2021) databases were used for predicting TFs binding to these promoters. A threshold of Q < 0.05 was used for filtering the results predicted by AnimalTFDB3.0, whereas a score >10 was considered for screening the results of the JASPAR database, and finally TFs predicted by both these databases were considered as the regulators of identified biomarkers.

#### 4.6.4. Prediction of Therapeutic Drugs Targeting the Potential Biomarkers

The drugs and molecular compounds targeting the biomarkers were predicted using the Comparative Toxicogenomics Database (CTD) (http://ctdbase.org/, accessed on 25 August 2021) for exploring potential treatment options for Group I PAH patients.

### 4.7. Verifying the Expression of Potential Biomarkers Using in Vivo and In Vitro Experiments

#### 4.7.1. Cell Culture and Hypoxia Culture Conditions

Human pulmonary microvascular endothelial cells (HPMECs) were obtained from Yu Bo Biotech company (Shanghai, China) and were maintained in an endothelial cell (EC) medium (ScienCell Research Laboratories, San Diego, CA, USA) containing 5 mg/mL penicillin/streptomycin and 10% FBS at 37 °C. HPMEC cells were exposed to hypoxic [74] (N_2_/O_2_/CO_2_ ratio = 94:1:5) or normoxic (O_2_/CO_2_ ratio = 21:5) conditions for 24 h.

#### 4.7.2. Quantitative Reverse Transcription PCR (qRT-PCR)

The mRNA expression levels of the potential biomarkers were first determined through qRT-PCR. A TRIzol reagent (Invitrogen, Carlsbad, CA, USA) was used for the isolation of total RNA from HPMECs. cDNA was prepared with the PrimeScript RT reagent kit (Vazyme, Nanjing, China) using the below-mentioned primer sequences. Subsequently, qRT-PCR was performed using the SYBR Green Realtime PCR Premix (Vazyme, Nanjing, China). The primer sequences used for the qRT-PCR experiment are provided in Table 6. The 2^−∆∆CT^ approach was used for determining the mRNA expression levels with GAPDH used as a reference.

#### 4.7.3. Human Lung Tissue Samples

We collected lung tissue samples from Group I PAH patients (*n* = 3 males of 35, 40, and 42 years, respectively; samples were collected during lung biopsy for diagnosis surgery) and controls (*n* = 3 males of 50, 58, and 65 years, respectively; para-carcinoma tissue samples were dissected during surgical resection of cancerous lung). The mean pulmonary artery pressure was 50 ± 6 and 20 ± 4 mmHg for Group I PAH patients and controls, respectively. The study was approved by the Ethics Committee of Zhongnan Hospital of Wuhan University and was carried out following the Declaration of Helsinki. Patients provided informed consent before sample collection.

#### 4.7.4. Immunohistochemistry (IHC)

Immunohistochemical staining was used to determine the expression of the potential biomarkers at the protein level. Lung tissue samples from Group I PAH patients and controls were processed by fixation, embedding, sectioning, and staining with rabbit antihuman PBRM1 polyclonal antibody (1:200, ABclonal, Oxfordshire, UK) and rabbit antihuman TXLNG polyclonal antibody (1:200, ABclonal) for detecting protein expression. Then, the samples were hybridized with biotinylated antirabbit secondary antibody and the avidin biotin enzyme complex (1:200, Abcam, MA, USA) staining was observed using an Olympus IX73 microscope.

### 4.8. Statistical Analyses

R packages and online software were used for statistical analyses. Normally distributed continuous data were compared by a *t*-test between groups. The statistical analysis was performed with a one-way ANOVA. A *p*-value < 0.05 was considered statistically significant.

## 5. Conclusions

Group I PAH represents an uncommon progressive disease with a high complexity, which is difficult to treat and can ultimately lead to death. In this study, diagnostic models using the lung tissue data from Group I PAH patients were compared through multiple machine learning algorithms, and finally, a new diagnostic model including PBRM1, CA1, and TXLNG genes was established. We performed a comprehensive analysis of the molecular biomarkers, including signaling pathway, PPI network, immune infiltration, regulatory TF network, potential therapeutic drug, and ferroptosis-related gene analyses. Finally, the expression of the biomarkers and a selected set of candidate genes were experimentally validated at the mRNA and protein levels.

## Figures and Tables

**Table 1:** Key candidate genes identified for Group I PAH.

Candidate Gene	Regulation	logFC	*p*-Value	AUC	AUC CI
*KLRF1*	down	−1.349514767	0.11706773	0.783	0.664–0.902
*CX3CR1*	down	−1.287895847	0.022492566	0.783	0.658–0.908
*TSPAN7*	down	−1.022431101	0.012950506	0.926	0.857–0.994
*AJAP1*	down	−1.118195957	0.036788191	0.848	0.744–0.953
*SOSTDC1*	down	−1.680530516	0.000676038	0.862	0.755–0.969
*S100A3*	down	−1.590859215	0.0016257	0.905	0.829–0.982
*C2orf40*	down	−1.066587667	0.019138646	0.838	0.727–0.95
*DLL4*	down	−1.070844547	0.03607353	0.879	0.79–0.907
*SCN4B*	down	−1.088543027	0.043459681	0.895	0.813–0.978
*ALAS2*	up	1.755557936	0.004588542	0.903	0.824–0.981
*CA1*	up	1.639222475	0.001211991	0.931	0.866–0.995
*TXLNG*	up	1.606123262	0.003413892	0.937	0.869–1
*LARS*	up	1.478386085	0.16211777	0.922	0.85–0.994
*CEP97*	up	1.473332937	0.037459459	0.893	0.802–0.984
*PBRM1*	up	1.156107034	0.015174985	0.966	0.923–1
*ACE2*	up	1.107381186	0.024049503	0.919	0.84–0.998
*VEPH1*	up	1.148259545	0.019732353	0.891	0.81–0.972
*ESF1*	up	1.139501155	0.023748417	0.908	0.825–0.99
*ZNF148*	up	1.040895681	0.033516829	0.937	0.876–0.998
*RSRC1*	up	1.103381776	0.03927137	0.96	0.916–1
*SECISBP2L*	up	1.067323553	0.044551819	0.903	0.822–0.984
*ZFX*	up	1.066420893	0.038982434	0.938	0.88–0.996
*EPHA4*	up	1.067115797	0.039949703	0.905	0.817–0.994
*HSP90AB3P*	up	1.145135459	0.01306175	0.92	0.844–0.997
*PPP1R9A*	up	1.013500448	0.039078627	0.924	0.861–0.987
*PARP14*	up	1.024736403	0.040732053	0.847	0.747–0.948
*ITSN2*	up	1.147534029	0.022053977	0.908	0.829–0.987
*PKP2*	up	1.167878389	0.013092511	0.824	0.72–0.929
*USP15*	up	1.206258609	0.009817843	0.933	0.868–0.998
*RSPO3*	up	1.189539709	0.010857124	0.881	0.78–0.982
*IQGAP2*	up	1.458016279	0.00580652	0.819	0.698–0.941
*IL13RA2*	up	1.350594967	0.00580652	0.819	0.698–0.941
*FKBP5*	up	1.296433874	0.006691228	0.827	0.718–0.936
*ZNF292*	up	1.278994973	0.00751151	0.881	0.791–0.971
*ANKRD50*	up	1.271971317	0.007723989	0.908	0.83–0.986
*RFC1*	up	1.247043964	0.016211777	0.946	0.893–0.998
*MACC1*	up	1.246789565	0.017027733	0.885	0.801–0.969
*TFEC*	up	1.32215869	0.008379207	0.918	0.843–0.982
*IGF1*	up	1.318199859	0.010011771	0.902	0.815–0.988
*FMO5*	up	1.2962632	0.010504888	0.792	0.674–0.909
*FGD4*	up	1.23373543	0.023974054	0.948	0.896–1
*MPP7*	up	1.245878219	0.04033993	0.902	0.817–0.986
*EHF*	up	1.485013582	0.002842598	0.919	0.845–0.993
*ZC3H13*	up	1.433143528	0.004006581	0.934	0.871–0.998
*TTN*	up	1.585014364	0.0055049	0.856	0.756–0.956
*CCDC88A*	up	1.014479213	0.045188545	0.866	0.775–0.957
*HSP90AA1*	up	1.145135459	0.01306175	0.889	0.804–0.974
*ROCK2*	up	1.145759039	0.0032232649	0.939	0.881–0.998

**Table 2:** Results for diagnostic value and accuracy of seven machine learning algorithms through a 5-fold cross-validation approach.

Nfold	1		2		3		4		5		Mean	
	AUC	Accuracy Rate	AUC	Accuracy Rate	AUC	Accuracy Rate	AUC	Accuracy Rate	AUC	Accuracy Rate	AUC	Accuracy Rate
RF	1.00	0.83	1.00	1.00	0.83	0.82	1.00	0.91	1.00	1.00	0.97	0.91
XGB	0.67	0.67	0.90	0.92	0.69	0.82	0.94	0.91	1.00	1.00	0.84	0.86
GBDT	1.00	0.92	1.00	0.92	1.00	0.91	1.00	0.91	1.00	1.00	1.00	0.93
ANN	0.89	0.89	1.00	0.92	0.94	0.91	1.00	1.00	1.00	1.00	0.97	0.93
DT	0.67	0.67	0.90	0.92	0.69	0.82	0.73	0.82	0.93	0.91	0.78	0.83
AdaBoost	0.83	0.92	0.80	0.83	0.69	0.82	1.00	1.00	1.00	1.00	0.97	0.91
MNB	0.94	0.92	1.00	1.00	0.75	0.91	0.94	0.91	0.93	0.91	0.91	0.93

nfold: N-fold cross-validation represented by numbers 1 through 5; AUC: Area under ROC curve; RF: random forest; XGB: extreme gradient boosting; GBDT: gradient boosting decision tree; ANN: artificial neural network; DT: decision tree; AdaBoost: adaptive boosting; MNB: multinomial naïve Bayes.

**Table 3:** Correlation between PAH biomarkers and immune cells.

Immune Cells	CA1		PBRM1		TXLNG	
	Correlation *R*	*p*-Value	Correlation *R*	*p*-Value	Correlation *R*	*p*-Value
Activated CD8 T cell	−0.32	0.01611	−0.37	0.00459	−0.36	0.00526
Central memory CD4 T cell	−0.26	0.05130	−0.32	0.01490	−0.35	0.00840
Effector memory CD4 T cell	0.33	0.01170	0.61	4.21 × 10^−7^	0.44	0.00061
Monocyte	−0.18	0.18123	−0.41	0.00147	−0.46	0.00035
Natural killer cell	−0.03	0.80401	0.19	0.15172	0.18	0.17243
Natural killer T cell	−0.25	0.05918	−0.31	0.01999	−0.29	0.03127
T follicular helper cell	−0.26	0.04738	−0.32	0.01399	−0.36	0.00554

Correlation between the biomarkers (first row) and immune cells (first column) is represented as *R* values and their significance as *p*-values. Positive and negative *R* values represent positive and negative correlations, respectively. The *Pearson correlation* method was used for the analysis.

**Table 4:** Potential therapeutic drugs targeting PAH biomarkers.

Biomarker Gene Symbol	Chemical	Interaction Action	Biomarker Gene Symbol	Chemical	Interaction Action
*PBRM1*	Acetaminophen	Increases	*TXLNG*	1-Methyl-3-isobutylxanthine	Increases
*PBRM1*	Antirheumatic agents	Increases	*TXLNG*	Aristolochic acid I	Decreases
*PBRM1*	Aristolochic acid I	Decreases	*TXLNG*	Aflatoxin B1	Decreases
*PBRM1*	Atrazine	Decreases	*TXLNG*	Atrazine	Other
*PBRM1*	Copper sulfate	Decreases	*TXLNG*	Bisphenol F	Increases
*PBRM1*	Cyclosporine	Increases	*TXLNG*	Copper sulfate	Increases
*PBRM1*	Doxorubicin	Other	*TXLNG*	Cylindrospermopsin	Increases
*PBRM1*	Epigallocatechin gallate	Decreases	*TXLNG*	Dexamethasone	Increases
*PBRM1*	Lactic acid	Decreases	*TXLNG*	Doxorubicin	Decreases
*PBRM1*	Methotrexate	Increases	*TXLNG*	Valproic acid	Increases
*PBRM1*	Methylmercuric chloride	Other	*TXLNG*	Ethyl methanesulfonate	Decreases
*PBRM1*	Plant extracts	Increases	*TXLNG*	Hydrogen peroxide	Other
*PBRM1*	Potassium chromate	Decreases	*TXLNG*	Indomethacin	Increases
*PBRM1*	Quercetin	Increases	*TXLNG*	Jinfukang	Decreases
*PBRM1*	Riddelliine	Decreases	*TXLNG*	Perfluoro-n-nonanoic acid	Increases
*PBRM1*	Sunitinib	Increases	*TXLNG*	Potassium chromate (VI)	Increases
*PBRM1*	Trichostatin A	Decreases	*TXLNG*	Sunitinib	Increases
*PBRM1*	TAK-243	Other	*TXLNG*	Tobacco smoke pollution	Increases
*PBRM1*	Valproic acid	Decreases	*TXLNG*	Tretinoin	Decreases
*CA1*	Acetaminophen	Decreases	*CA1*	Bicarbonates	Other
*CA1*	Acetazolamide	Decreases	*CA1*	Bromates	Decreases
*CA1*	Aflatoxin B1	Other	*CA1*	Butylated hydroxyanisole	Decreases
*CA1*	Amides	Decreases	*CA1*	Candesartan	Decreases
*CA1*	Anthocyanins	Decreases	*CA1*	Carbonates	Decreases
*CA1*	Benzolamide	Decreases	*CA1*	Chalcone	Decreases
*CA1*	Chalcone epoxide	Decreases	*CA1*	Crown ethers	Other
*CA1*	Chloric acid	Decreases	*CA1*	Dimethylamines	Decreases
*CA1*	Cobalt	Decreases	*CA1*	Ethoxzolamide	Decreases
*CA1*	Cobaltous chloride	Decreases	*CA1*	Flavonoids	Decreases
*CA1*	Coumarin	Decreases	*CA1*	Guaiacol	Decreases
*CA1*	Crown compounds	Decreases	*CA1*	Indomethacin	Decreases
*CA1*	Iodates	Decreases	*CA1*	Mercury	Decreases
*CA1*	Irbesartan	Decreases	*CA1*	Methazolamide	Decreases
*CA1*	Lactic acid	Increases	*CA1*	Methylamines	Decreases
*CA1*	Lead	Decreases	*CA1*	Nitric acid	Decreases
*CA1*	Malvidin-3-glucoside	Decreases	*CA1*	Oryzalin	Decreases
*CA1*	Malvin	Decreases	*CA1*	pelargonidin-3-glucoside	Decreases
*CA1*	Perchlorate	Decreases	*CA1*	Silychristin	Decreases
*CA1*	Phenylephrine	Decreases	*CA1*	Sodium arsenite	Decreases
*CA1*	Phenylthiourea	Decreases	*CA1*	Sodium metasilicate	Decreases
*CA1*	Potassium periodate	Decreases	*CA1*	Sulfamic acid	Decreases
*CA1*	Propofol	Decreases	*CA1*	Sulfates	Decreases
*CA1*	Pyrimidines	Decreases	*CA1*	Sulfonamides	Decreases
*CA1*	Rifampin	Decreases	*CA1*	Synephrine	Decreases
*CA1*	Rosiglitazone	Other	*CA1*	Thiazolidines	Decreases
*CA1*	Thiones	Decreases	*CA1*	Triazoles	Decreases
*CA1*	Thiophenes	Decreases	*CA1*	Tungstate	Decreases
*CA1*	Thiosemicarbazide	Decreases	*CA1*	Vanadates	Decreases
*CA1*	Thiourea	Decreases	*CA1*	Vanillin	Decreases
*CA1*	Tobacco smoke pollution	Decreases	*CA1*	Voriconazole	Decreases
*CA1*	Topiramate	Decreases	*CA1*	Zonisamide	Decreases

**Table 5:** Correlation between biomarkers and ferroptosis-related genes.

Biomarker Gene	Fer-Related Gene	Correlation *R*	*p*-Value	Biomarker Gene	Fer-Related Gene	Correlation *R*	*p*-Value
*PBRM1*	*BECN1*	0.869423423	1.76 × 10^−18^	*TXLNG*	*ZEB1*	0.773220309	1.81 × 10^−12^
	*HMGB1*	0.815007386	1.20 × 10^−14^		*BECN1*	0.731996746	9.84 × 10^−11^
	*SP1*	0.757174898	9.40 × 10^−12^		*HMGB1*	0.724502669	1.88 × 10^−10^
	*ZEB1*	0.728618544	1.32 × 10^−10^		*LPCAT3*	−0.705414565	8.97 × 10^−10^
	*IREB2*	0.707501483	7.60 × 10^−10^		*RIPK1*	0.699642666	1.40 × 10^−9^
	*PLIN2*	0.657847236	2.70 × 10^−8^		*PRDX6*	0.61934584	2.80 × 10^−7^
	*SLC3A2*	−0.633598312	1.22 × 10^−7^		*SP1*	0.60633034	5.77 × 10^−7^
	*LPCAT3*	−0.61500838	3.58 × 10^−7^		*IREB2*	0.604841956	6.26 × 10^−7^
	*RIPK1*	0.610161027	4.68 × 10^−7^		*YWHAE*	−0.602218341	7.20 × 10^−7^
*CA1*	*PLIN2*	0.629426281	2.29 × 10^−5^	*CA1*	*IREB2*	0.696001081	8.73 × 10^−5^

**Table 6:** Primers used for qRT-PCR.

Gene Name	Forward Primer (5′-3′)	Reverse Primer (5′-3′)
*PBRM1*	AGGAGGAGACTTTCCAATCTTCC	CTTCGCTTTGGTGCCCTAATG
*CA1*	CTGACAGCTACAGGCTCTTTC	CTACGTGAAGCTCGGCAGAAT
*TXLNG*	ATCCATCAAAGCGCCATCAAAGCG	ACAAATAAAGCAATAGCATCACAA
*IGF1*	AAGCCTACAAAGTCAGCTGC	GGTCTTGTTTCCTGCACTT
*ACE*	TCCTATTCCCGCTCATCT	CCAGCCCTTCTGTACCATT
*RSPO*	CAGCCATAACTTCTGCACCA	AGAGCTGCTGCTTCTTGGAG
*C2orf40*	GGTACCAGCAGTTTCTCTACATG	CAGCGTGTGGCAAGTCATGGTTAGT
*TSPAN7*	CTCATCGGAACTGGCACCACTA	CCTGAAATGCCAGCTACGAGCT
*SCN4B*	CAACAGCAGTGACGCATTCAA	CTCCTTAGTAGAGCCTACCAGAG
*IREB2*	GCGATTTCCAGGCTTGCTTA	GTTTAACACGCAGACCAGCT
*LPCAT3*	AGCCTTAACAAGTTGGCGAC	TGCCGATAAAACAAAGCAAA
*BECN1*	AGGAACTCACAGCTCCATTAC	AATGGCTCCTCTCCTGAGTT
*ZEB1*	AAACTCGAGTACTTCAATTCCTCGGTATTG	AAATCTAGACACACTGTTCTACAGTCCAA
*HMGB1*	ATATGGCAAAAGCGGACAAG	AGGCCAGGATGTTCTCCTTT
*SLC3A2*	ACCCCTGTTTTCAGCTACGG	GGTCTTCACTCTGGCCCTTC
*PLIN2*	CTGTCTACCAAGCTCTGCTC	CGATGCTTCTCTTCCACTCC
*SP1*	GTGGAGCAACATCATTGCTG	GCCACTGGTACATTGGTCACAT
*RIPK1*	AGGCTTTGGGAAGGTGTCTC	CGGAGTACTCATCTCGGCTTT
*PRDX6*	TTTCAATAGACAGTGTTGAGGATCA	CGTGGGTGTTTCACCATTG
*YWHAE*	CTAACACTGGCGAGTCCAAGGT	GTAAGCCACG AGGCTGTTCTCT
*GAPDH*	CAATGACCCCTTCATTGACC	TTGATTTTGGAGGGATCTCG

## Data Availability

Data for this study are available from the corresponding author upon reasonable request.

## Supplementary Materials

The supporting information can be downloaded at: https://www.mdpi.com/article/10.3390/ijms24098050/s1.
